# RETRACTED: Liu et al. Andrographolide Alleviates Oxidative Damage and Inhibits Apoptosis Induced by IHNV Infection via CTSK/BCL2/Cytc Axis. *Int. J. Mol. Sci.* 2024, *25*, 308

**DOI:** 10.3390/ijms26010208

**Published:** 2024-12-30

**Authors:** Qi Liu, Linfang Li, Jingzhuang Zhao, Guangming Ren, Tongyan Lu, Yizhi Shao, Liming Xu

**Affiliations:** 1 Heilongjiang River Fisheries Research Institute, Chinese Academy of Fishery Sciences, Harbin 150070, China; 2 Key Laboratory of Aquatic Animal Diseases and Immune Technology of Heilongjiang Province, Harbin 150070, China

The journal retracts the article “Andrographolide Alleviates Oxidative Damage and Inhibits Apoptosis Induced by IHNV Infection via CTSK/BCL2/Cytc Axis” [1] cited above.

Following publication, the authors contacted the Editorial Office regarding concerns relating to the presence of scientific errors that impact the overall validity of the findings presented in this study [1].

During a review of the data, the authors identified errors within the data that indicated that the results relating to the cytotoxicity of andrographolide were incorrect and irreproducible. Adhering to our standard procedure, the Editorial Board evaluated the concerns raised and agreed with the authors that the overall findings of this study could not be relied upon. As a result, the Editorial Office, Editorial Board and authors have decided to retract this article [1], as per MDPI’s retraction policy (https://www.mdpi.com/ethics#_bookmark30).

This retraction was approved by the Editor-in-Chief of the *International Journal of Molecular Sciences*.

The authors agreed to this retraction.

## References

[1] Liu Q., Li L., Zhao J., Ren G., Lu T., Shao Y., Xu L. (2024). RETRACTED: Andrographolide Alleviates Oxidative Damage and Inhibits Apoptosis Induced by IHNV Infection via CTSK/BCL2/Cytc Axis. Int. J. Mol. Sci..

